# Determination of the breeding value of collection chickpea (Cicer arietinum L.) accessions by cluster analysis

**DOI:** 10.18699/VJ20.617

**Published:** 2020-05

**Authors:** N.A. Vus, L.N. Kobyzeva, O.N. Bezuglaya

**Affiliations:** Plant Production Institute nd. a. V.Ya. Yuryev of National Academy of Agrarian Sciences of Ukraine, Kharkiv, Ukraine; Plant Production Institute nd. a. V.Ya. Yuryev of National Academy of Agrarian Sciences of Ukraine, Kharkiv, Ukraine; Plant Production Institute nd. a. V.Ya. Yuryev of National Academy of Agrarian Sciences of Ukraine, Kharkiv, Ukraine

**Keywords:** chickpea, cluster analysis, genetic resources, sources of valuable traits, breeding, нут, кластерный анализ, генетические ресурсы, источники ценных признаков, селекция

## Abstract

Assessment of the genetic resources of chickpea (Cicer arietinum L.) in a zone that is atypical for its cultivation (eastern forest-steppe of Ukraine) gives an opportunity to identify valuable starting material for priority breeding areas. The article presents the results of a cluster analysis on chickpea accessions from the National Center for Plant Genetic Resources of Ukraine (NCPGRU) for a set of agronomic characteristics. In 2005–2017, 653 chickpea accessions from the NCPGRU’s core collection were studied: 369 kabuli accessions and 284 desi accessions. One hundred and fifty two sources of valuable traits were identified for 11 parameters: drought tolerance, resistance to Ascochyta leaf and pod spot, early ripening (vegetation period length), yield, performance, number of productive pods and seed number per plant, response to nitrogenization, protein content, seed size, and cooking quality. These accessions (77 kabuli accessions are light-colored and 75 desi ones are dark-colored) were grouped by a set of valuable economic characteristics using cluster analysis with the Euclidean distance as a measure. The study showed that this sample consisted of 4 clusters. Cluster 1 contained mainly kabuli accessions with optimal combinations of valuable traits: drought tolerance, resistance to Ascochyta leaf and pod spot, large seeds, high yield capacity and performance, pod and seed numbers as well as protein content in seeds. This cluster includes standards and most of reference varieties, which are well-adapted to the conditions of the eastern forest-steppe of Ukraine. The accessions of cluster 2 are characterized by high resistance to Ascochyta leaf and pod spot, late ripening, small seeds, low protein content, moderate response to nitrogenization, high performance attributed to a large number of productive pods and seeds per plant. Most of the accessions of this cluster are small-seeded late-ripening kabuli accessions. Cluster 3 consists of 3 accessions, which have large seeds and high protein content in them, give moderate yields, are highly responsive to nitrogenization and poorly resistant to Ascochyta leaf and pod spot. Cluster 4 comprises mainly desi accessions (63 %), which are mid-ripening, with small seeds, low performance, moderate yield capacity, medium protein content, poor cooking quality, moderate resistance to Ascochyta leaf and pod spot, and low drought tolerance. Representatives of this cluster are predominantly sources of one trait and may have restricted application in specialized breeding programs. Based on the data obtained, we concluded that the accessions of cluster 1 were preferable in breeding programs to develop chickpea varieties for the foreststeppe zone.

## Abstract (ru)

Оценка генетических ресурсов нута (Cicer arietinum L.) в нетипичной для выращивания зоне (восточная часть лесостепи Украины) дает возможность выделить ценный исходный материал для приоритетных направлений селекции. В статье представлены результаты кластерного анализа образцов нута Национального центра генетических ресурсов растений Украины (НЦГРРУ) по комплексу агрономических характеристик. В период 2005–2017 гг. были изучены 653 образца базовой коллекции нута НЦГРРУ: 369 об- разцов морфотипа kabuli и 284 – desi. Выделены 152 источника ценных признаков по 11 показателям: засухоустойчивости, устойчивости к аскохитозу, скороспелости (длительности вегетационного периода), урожайности, продуктивности, количеству продуктивных бобов, количеству семян с одного растения, реакции на нитрагинизацию, содержанию белка, крупности семян, разваримости. Эти образцы (77 типа kabuli – светлоокрашенные и 75 desi – темноокрашенные) были сгруппированы по комплексу ценных хозяйственных признаков с помощью кластерного анализа методом евклидовых расстояний. Результаты исследования показали, что представленная выборка состоит из четырех кластеров. В кластере 1 преобладают образцы типа kabuli с оптимальным сочетанием ценных признаков: засухоустойчивость, устойчивость к аскохитозу, крупносемянность, высокая урожайность и продуктивность, количество бобов и семян, содержание белка в семенах. В этот кластер вошли стандарты и большинство эталонов, которые характеризуются высокой адаптивностью к условиям восточной части лесостепи Украины. Образцы кластера 2 отличаются высокой устойчивостью к аскохитозу, позднеспелостью, мелкосемянностью, низким содержанием белка, средней реакцией на нитрагинизацию и высокой продуктивностью за счет большого количества продуктивных бобов и семян с одного растения. Большая часть образцов этого кластера – мелкосемянные позднеспелые образцы типа kabuli. Кластер 3 состоит из трех образцов, отличающихся высокой крупностью семян, средним уровнем урожайности, высокой реакцией на нитрагинизацию и повышенным содержанием белка при низкой устойчивости к аскохитозу. Кластер 4 объединяет преимущественно образцы морфотипа desi (63 %), среднеспелые со средними показателями урожайности, содержания белка и устойчивости к аскохитозу, с невысокой крупностью семян и продуктивностью, низкой разваримостью и засухоустойчивостью. Представители этого кластера – преимущественно источники одного признака – и могут иметь узкое применение в специализированных селекционных программах. На основе полученных данных предлагается приоритетное использование образцов первого кластера в селекционных программах по созданию сортов нута для лесостепной зоны.

## Introduction

The world chickpea production is growing with every passing year, especially in arid regions, where it is the main source of protein for the population. Lack of varieties suitable for cultivation in different geographical zones of the country, which would combine high seed quality and resistance to bio- and abiotic factors, is the main limitation that hinders the spread of chickpea in Ukraine. The leading countries for chickpea cultivation have already gone this way, have develop and continue to develop plastic varieties, which allows expanding their cultivation regions.

Exploration of genetic diversity is very useful, when one works with genetic resources and for breeding programs, and includes labeling, identification and/or removal of duplicates in the gene pool and creation of core collections (Aliu et al., 2016).

Chickpea is a cheap source of high-quality protein in the diet of millions of people, which is a good alternative to animal protein for a balanced nutrition. The quality of chickpea protein is second only to milk protein. It is the second most important grain legume in the world, and in some parts, such as the Indian subcontinent, the first (Singh et al., 2015). Two main chickpea types are distinguished in the crop: desi and kabuli. Desi chickpea seeds are small, angular with dark seed coats; kabuli seeds are relatively large, smooth, yellow, yellow-pink or cream in color.

Kabuli chickpea seeds are considered more valuable due to high contents of protein, dietary fiber, complex carbohydrates and minerals. Both decorticated (removal of the seed coat) and non-decorticated chickpea seeds are used. Use without decortication involves boiling, frying or grinding to a paste (for example, in hummus manufacture). Desi chickpea is used both as split beans (dhal) and as flour (besan). Chickpea flour mixed with wheat or rice flour is used for making bread (chapati) and in confectionery (Tripathi et al., 2012). High drought tolerance plays an important role in the significant spread of chickpea. This feature of chickpea allows its growing in risky farming areas with limited precipitation and obtaining high yields of valuable dietary protein. With the expansion of arid zones and extension of rainless periods, this feature of the crop widens the prospects for its cultivation. However, to increase the sown acreage, it is necessary to develop new chickpea varieties that are adapted to specific conditions.

Food and Agriculture Organization (FAO) informs that the implementation of innovative breeding programs has increased the total yield of chickpea from 0.71 t/ha in 1996 to 0.96 t/ha in 2014 (FAOSTAT, 2016).

In the last two years, the chickpea production in Ukraine has grown from 6.5 thousand tons to 19.2 thousand tons due to introduction of domestic varieties, such as Pamiat, Triumf, Budzhak, and Odissei, with a potential yield of 1.8–2.3 t/ha (Kernasyuk, 2018). Ukrainian breeders were able to achieve such results owing to active involvement of the chickpea gene pool, which is studied in the National Center for Plant Genetic Resources of Ukraine (NCPGRU, Kharkiv). The NCPGRU’s collection of chickpea comprises 1,897 accessions, with foreign varieties accounting for 91 % of the collection. One hundred and thirty four accessions are of Ukrainian origin; 38 of them are breeding varieties. FAO regards the NCPGRU’s chickpea collection as one of the most important in the world by volume and diversity. All accessions are studied for three years and evaluated for phenological and morphological characteristics, disease resistance, quality and chemical composition of seeds, and, as a result of this work, sources of valuable traits are identified for further breeding. Based on results, working, trait, genetic and other collections are formed (The Second Report..., 2010).

To develop new varieties, it is important to use well-studied and selected starting material. In addition, numerous domestic and foreign studies show that breeding varieties have a narrow genetic base, despite a wide assortment of chickpea accessions stored in genebanks of different countries (Akinina, Popov, 2012; Khamassi et al., 2012; Benzohra et al., 2013;Vus et al., 2017a). Expansion of the genetic base by involving new parents in crossing is an important mechanism in breeding, but this material should be well-studied and adapted to a specific climatic zone.

Work with a collection of plant genetic resources implies evaluating large numbers of accessions for a wide range of different qualitative traits, which was applied to explore collections of corn (Kroonenberg et al., 1995), rice (Nandini et al., 2017), lentil (Shikhalieva et al., 2018), chickpea (Malik et al., 2014) and other crops. For the convenience of breeding, different genotypes are grouped into clusters according to the on genetic diversity, and the degree of genetic divergence between them is assessed. For this purpose, cluster analysis is one of the most acceptable tools to assess the relative contribution of different traits – constituents to the total diversity, to quantify the degree of divergence and to choose genetically diverse parents to generate desirable recombinants. This was successfully done by N. Gupta et al., who studied 20 grape accessions for 9 yield traits (Gupta et al., 2017) and by E.J. Oliveira et al., who analyzed the cassava genebank in Brazil (Oliveira et al., 2016). The use of this method for evaluating breeding material at the early stages of breeding can accelerate the development of new varieties (Vilchinskaya et al., 2017). It is used to analyze agronomic, phenological, and morphological features in different crops, both to evaluate hybridization results and to assess the gene pool diversity (Motavassel, 2013). To solve this problem, researchers use different variants of cluster analysis. For example, evaluating 11 cotton accessions, L.F. Araújo et al. concurrently applied several variants (single-link method, complete link method, median, average linkage within the cluster and average linkage between the clusters) and found that the average linkage between the clusters was the most effective for their purpose (Araújo et al., 2014).

Cluster analysis is widely used in the breeding practice for assessment of different crops and comparison of their parameters. G. Evgenidis et al. applied this method, working with tomato starting material (Evgenidis et al., 2011). M. Khodadadi used the Euclidean distance method and Ward’s method (Khodadadi et al., 2011) for assessing the genetic diversity and selection of parental pairs from 36 winter wheat accessions. The Euclidean distance method was also used by A. Subramanian and N. Subbaraman to group 38 corn accessions by 25 traits, which allowed them to identify the most distant pairs for crossing and showed that the geographical origin was not associated with expression of the studied traits (Subramanian, Subbaraman, 2010). P.M. Kroonenberg et al. clearly demonstrated the effectiveness of cluster analysis approaches for investigating collections of corn resources (Kroonenberg et al., 1995).

To assess the genetic diversity of wheat, B. Mecha et al. used cluster analysis and principal component method (Mecha et al., 2017). V. Nandini used the K-average method to assess 14 agronomic traits in rice accessions (Nandini et al., 2017). To select starting material for breeding for the quality of green beans, the Mahalanobis method was used (Haralayya et al., 2017). A. Kahraman et al. used the Euclidean distance method, which enables one to assess the similarity between accessions and identify groups of similar accessions, to group 35 bean accessions (Kahraman et al., 2014). The same method was used to analyze the results of studying major economically valuable traits in lentil accessions (Shikhalieva et al., 2018). S.R. Malik used cluster analysis to evaluate 113 desi chickpea accessions for 11 agronomic traits, which enabled him to identify a group of accessions with a set of traits combining the most valuable genotypes for further breeding (Malik et al., 2014). S. Aliu et al. investigated the qualitative composition of chickpea seeds and its association with yield indicators (Aliu et al., 2016).

Cluster analysis methods allow comparing various numbers of accessions: from 6 (Kayan, Adak, 2012) to over 300 (Naghavi et al., 2012) by different quantity and quality of characteristics under investigation: both qualitative and quantitative ones. Scientists from countries where chickpea is an important crop have used cluster analysis to evaluate genetic or selection material. For cluster analysis, both phonological/ morphological and genetic features are used (Hajibarat et al., 2014; Aggarwal et al., 2015). For comparison of diverse agronomically valuable traits, such as yield capacity, resistance to diseases, growing period length and seed quality, the Euclidean distance method, which enables one to identify the hierarchical structure among the studied accessions, group them by a set of traits and select the most promising pairs for crossing, is most often used to study chickpea accessions. This method allows evaluating both similarity and distinction between accessions and characterizes the degree of expression of a trait under investigation (Syed et al., 2012; Malik et al., 2014). This makes it possible to distinguish the most distant accessions for crossing, which can positively affect the heterosis effect.

Cluster analysis allows one to compare accessions, using genetic markers, and characterize expression levels of the studied traits, for example, in inbred corn lines or in offspring from one cross (Subramanian, Subbaraman, 2010; Shrestha, 2016). K. Khamassi et al.’s studies proved that clustering by morphological characteristics was close to genotypic clustering by SSR markers (r = 0.554; p = 0.001). This makes it possible to conduct an effective selection of parental pairs for crossing based on assessments of morphological characteristics only (Khamassi et al., 2012).

Our purpose was to group chickpea accessions using cluster analysis and to select sources from clusters that have several valuable economic characteristics for further breeding.

## Materials and methods

Six hundred and fifty three chickpea accessions from the core collection of the NCPGRU were taken as the test material for studies conducted in 2005–2017. The studied accessions were represented by two non-taxonomic groups: 369 kabuli accessions (light-seeded – white, cream, beige) and desi 284 accessions (dark-seeded – red, brown, green, black and other colors). Geographically the studied accessions originate from 20 countries. The majority of the kabuli accessions are from Ukraine (21 %); 20 % – from India; 13 % – from Syria; 11 % – from Afghanistan; and 10 % – from Iran. The vast majority of desi accessions originate from India (46 %); 12 % – from Canada; 7 % – from Syria; and 7 % – from Ukraine. Biologically, the accessions are represented by old and modern commercial domestic and foreign varieties and lines. The vast majority of the studied assortment were breeding lines: kabuli – 63 % and desi – 77 %.

Eleven parameters were assessed: drought tolerance, resistance to Ascochyta leaf and pod spot, early ripening (vegetation period length), yield capacity, performance, number of productive pods and number of seeds per plant, response to nitrogenization, protein content, seed size, and cooking quality.

The field experiments were conducted in scientific crop rotation 1 in the collection nursery of the Laboratory of Genetic Resources of Grain Legumes and Groat Crops of the Plant Production Institute named after V.Ya. Yuriev of National Academy of Agrarian Sciences of Ukraine, which is located in the Kharkiv district of Kharkiv region in the North-East of the Left-Bank forest-steppe of Ukraine. Farmingtechniques were conventional for grain legume cultivation in this zone. Winter wheat was the forecrop. Chickpea was sown with manual planters; the record area was 1 m2; the sowing scheme: 10 × 30 cm. Check varieties were sown every other 20 collection accessions. Variety Rozanna (Ukraine) was taken as the check variety for kabuli-type; Krasnokutskiy 123 (Russia) – for desi-type.

The collection chickpea accessions were studied in accordance with “Methodical Recommendations for Studies into Grain Legume Genetic Resources of the All-Union Research Institute of Plant Breeding” (1975) and “Methodological Recommendations for Studies into Grain Legume Genetic Resources” (Kobyzeva et al., 2016). The accessions were categorized by economic and biological characteristics using the classifier of the genus Cicer L. (Bezuglaya et al., 2012). The collection accessions were assessed for resistance to Ascochyta leaf and pod spot in accordance with “Methodical Guidelines for Studies of Resistance of Grain Legumes to Diseases” (1976). Provocative background was used to simulate epiphytoties of Ascochyta leaf and pod spotin 2005 and 2016 (Kosenko, Bezuglaya, 2006; Bezuglaya et al., 2007; Vus et al., 2017a; Vus, Kobyzeva, 2018). The average scores for the epiphytotic years were used for the calculation matrix.

Drought tolerance was assessed by tolerance indices developed by foreign researchers (Fisher, Maurer, 1978; Rosielle, Hamblin, 1981; Bouslama, Schapaugh, 1984; Gavuzzi et al., 1997; Ribaut, Poland, 1999; Yücel, Mart, 2014). Previously we had evaluated drought tolerance of chickpea accessions using drought tolerance indices (Vus et al., 2017b) and developed a rating scale for assessing drought tolerance. Exceedance of median for each individual index is defined as one point. The total points give a seven-point score: from 0 to 7 points, where 0 – no exceedance of median for any index, 7 – exceedance of median for seven indices. Thus, in this work, we used the following drought tolerance scale: 0 points – the accession is very susceptible to drought, 7 – the accession is drought tolerant.

Response of the accessions to pre-sowing nitrogenization of seeds was studied in plots of 2 m2 without repetitions; the sowing scheme was 10 × 30 cm. The accessions were sown within the optimum timeframe, as described in (Didovich et al., 2010). Seeds were treated with rhizobophyte based on Mesorhizobium cicero (strain 065) immediately before sowing. The control was sown without seed treatment with rhizobophyte. The accessions’ responses to nitrogenization were expressed as a percentage increase in the yield related to the control.

The protein content in seeds was determined by Kjeldahl digestionin the Laboratory of Genetics, Biotechnology and Guality of the Plant Production Institute named after V.Ya. Yuriev of NAAS (Yermakov, 1987). The cooking quality was determined in accordance with “Methodological Recommendations. Technological Evaluation of Pea, Lentil, and Bean Grain” (Komarov, Proreshneva, 1992).

As a result of evaluating 653 accessions of the NCPGRU’s chickpea core collection by 11 parameters, 152 sources of valuable traits were identified: 77 kabuli and 75 desi accessions. These accessions were further used for grouping by the Euclidean distance method.

The experimental data were statistically processed by calculus of variations, analysis of variance and cluster analysis in Microsoft Excel (license No. 44208338, release date 06.27.2008) and Statistics 6.0 (license No. BXXR502C631 824NET3).

## Results

The primary differentiation of 152 accessions identified two clusters: A and B with similar numbers of accessions (78 and 74, respectively) (see the Figure). Most of the accessions in cluster A belong to kabuli-type (63 %), unlike cluster B, where 62 % of the accessions are desi. In this study, the types were only defined by the seed coat color: kabuli – light-colored and desi – dark-colored, which allowed us to establish relationships between valuable economic traits and seed color.

**Fig. 1.**
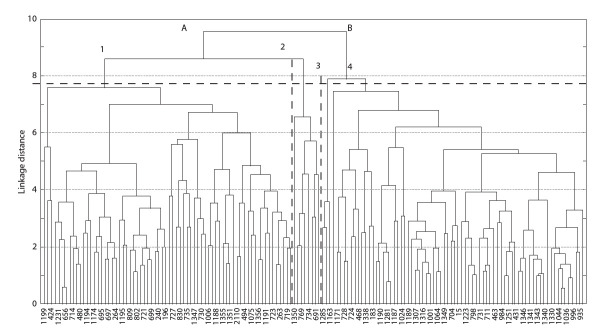
Clustering of chickpea (Cicer arietinum L.) accessions by a set of valuable economic characteristics using the Euclidean distance method.

Further, each of the primary clusters was divided into two unequal secondary clusters: the first cluster (A) – into cluster 1 (70 accessions) and cluster 2 (8 accessions) and the second cluster (B) – into cluster 3 (3 accessions) and cluster 4 (71 accessions) (Table).

**Table 1.**
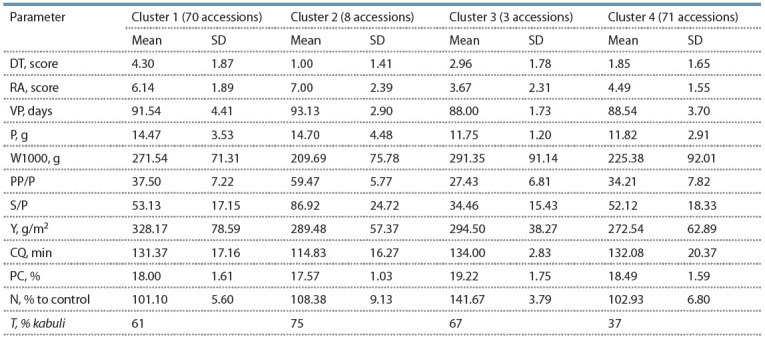
Distribution of the chickpea accessions between clusters Note. DT – drought tolerance; RА – resistance to Ascochyta leaf and pod spot; VP – vegetation period; P – performance; W1000 – 1000-seed weight; PP/P – number of productive pods per plant; S/P – number of seeds per plant; Y – yield; CQ – cooking quality; PC – protein content; N – nitrogenization; Т – type; SD – standard deviation.

Cluster 1 consists of 70 accessions, of which 43 belong to kabuli-type. These accessions optimally combine 8 of the 11 traits under investigation: drought tolerance (4.30 points), resistance to Ascochyta leaf and pod spot (6.14 points), seed size (271.54 g/1000 seeds), high yield (328.17 g/m2), performance (14.47 g/plant), the numbers of pods (37.50) and seeds (53.13), protein content in seeds (18.00 %). This cluster includes the check varieties for the kabuli- and desi-types: Rozanna (Ukraine) and Krasnokutskiy 123 (Russia). It also includes the NCPGRU’s reference accessions for resistance to Ascochyta leaf and pod spot (UD0500196 (Azerbaijan), UD0500264 (Ukraine) and UD0500240 (Syria)); for high palatability (UD0500417 (Ukraine)); and for suitability for mechanized harvesting (UD0500444 (Ukraine)). The accessions of this cluster are highly adapted to the conditions of the eastern forest-steppe of Ukraine and the most promising material for breeding programs to develop varieties with several useful features.

Cluster 2 is represented by eight accessions; six of them are of kabuli-type, and two – of desi-type. The accessions of this cluster are characterized by high resistance to Ascochyta leaf and pod spot (7.00 points), late ripening (vegetation period = 93.13 days), small seeds (1000-seed weight = 209.69 g), low protein content (17.57 %), moderate response to nitrogenization (108.38 % related to the control), and high performance (14.70 g/plant) attributed to the large numbers of productive pods (59.47) and of seeds (86.92) per plant. All the six kabuli accessions of this cluster are small-seeded, late-ripening and the most resistant to Ascochyta leaf and pod spot (Reddy, Singh, 1984). These are local accessions from Moldova (UD0500691, UD0500692, UD0500734, and UD0500762), Russia (UD0500769) and India (UD0501256). In addition, this cluster contains reference accessions of desi-type for the traits of “high number of seeds” and “high number of productive pods per plant”: UD0500022 (Georgia) and UD0501350 (India).

Cluster 3 has only three accessions: two of them belong to kabuli-type (UD0501163 (Ukraine) and UD0501268 (India)) and one accession is a desi one (UD0501285 (Syria)). The accessions of this cluster are characterized by large seeds (1000-seed weight = 291.35 g), moderate yield (294.50 g/ m2), strong response to nitrogenization (141.67 % related to the control), increased protein content (19.22 %), and low resistance to Ascochyta leaf and pod spot (3.67 points).

Cluster 4 mainly comprises desi accessions (63 %), which are characterized by mid-ripening (vegetation period = 88.54 days), small seeds (1000-seed weight = 225.38 g), low performance (11.82 g), moderate yield (272.54 g/m2), medium protein content (18.49 %), poor cooking quality (132.08 min), moderate resistance to Ascochyta leaf and pod spot (4.49 points), and low drought tolerance (1.85 points). The representatives of this cluster are mainly sources of one trait and can have only narrow application in specialized breeding.

## Discussion

The number of seeds and pod weight per plant are determining factors of the chickpea plant performance (Zali et al., 2011; Kazydub et al., 2015). Therefore, when selecting for performance, one should pay attention to these traits. In our study, few accessions (8) of cluster 2 produce the maximum numbers of productive pods and seeds per plant, and they have low indices of drought tolerance. At the same time accessions of cluster 1 with the maximum yield and above-average performance constituent in combination with resistance to Ascochyta leaf and pod spot and drought are of high value for breeding. Parameters such as performance, plant height, protein content and the pod number per plant are closely interrelated and positively correlate with yield (Kayan, Adak, 2012). In our studies, such characteristics were intrinsic to accessions of clusters 1 and 2.

Many researchers found that the growing period length, plant height, the numbers of branches and seeds per plant are mainly controlled by additive genes, directly and strongly correlating with the phenotypic trait of seed yield (Syed et al., 2012). Therefore, such traits-oriented breeding can be effective for increasing yield capacity. Stepwise regression analysis showed that the number of seeds per plant and 1000-seed weight accounted for 96 % of the total change in the yield. Therefore, the chickpea yield can be improved by choosing an idiotype having larger numbers of secondary and primary branches, larger numbers of pods and seeds per plant and a higher1000-seed weight of (Zali et al., 2011). We observed that the largest seeds were produced by accessions of cluster 3, however, they had the fewest productive pods and seeds per plant, while accessions of cluster 1, though having slightly smaller seeds than the average size, had larger numbers of seeds and productive pods. Therefore, priority should be given to accessions of cluster 1 in breeding for yield.

Often, researchers in chickpea study accessions of a single type A. Taleei studied desi accessions in Iran (Taleei, Shaabani, 2016), S.R. Malik – in Pakistan (Malik et al., 2014). M. Aarif (Aarif et al., 2017) and S. Aliu (Aliu et al., 2016) studied kabuli accessions in India and in Kosovo, respectively. We evaluated accessions of two types under the same conditions, and they were clearly differentiated in two primary clusters, A and B, and then in clusters 1 and 4, where the overwhelming majority of accessions were concentrated (70 and 71, respectively), kabuli accessions prevailed in cluster 1, and desi ones – in cluster 4. The involvement of accessions of different types from distant clusters in breeding will allow using seed color as a marker and expanding the genetic base of breeding varieties.

Large-seeded varieties are usually more susceptible to environmental conditions, therefore, selection of accessions and development of starting material with a high 1000-seedweight that are tolerant to biotic factors is important for breeding (Gridnev et al., 2012). To obtain breeding material with increased resistance to Ascochyta leaf and pod spot and several valuable traits, it is advisable to cross large-seeded accessions cluster 3 with accessions of clusters 1 and 2, which are highly-resistant to Ascochyta leaf and pod spot.

The systematic selection of parental pairs used in modern breeding upon crossing, the principles of which had been shaped by N.I. Vavilov (1967) and further developed by scientists from different countries (Serebrovskiy, 1969; Vural, Karasu, 2007; Aarif et al., 2017; Haralayya et al., 2017), shows that genotypes from clusters with a maximum distance between them can be used as parents in breeding to generate varieties with combining high yields and good seed quality, especially when accessions of different eco-geographical origin are crossed.

## Заключение

Cluster analysis is an effective method for evaluating a large number of collection chickpea accessions for several parameters, which enables one to select parental pairs for different breeding programs. Starting material with a set of features was selected and grouped into 4 clusters. Accessions of cluster 1 are preferable for breeding chickpea varieties adapted to the eastern forest-steppe of Ukraine. Accessions of cluster 2 are important in breeding for resistance to Ascochyta leaf and pod spot and high performance. Accessions of cluster 3 (UD0501163 (Ukraine), a reference accession of positive response to nitrogenization, with large seeds and high protein content in seeds; UD0501268 (India) with strong response to nitrogenization and moderate resistance to Ascochyta leaf and pod spot; and UD0501285 (Syria) with large seeds, moderate resistance to Ascochyta leaf and pod spot) are highly valuable for developing commercial chickpea varieties, however, they require augmenting their adaptive features. Accessions of cluster 4 are sources of one trait may have narrow application in specialized breeding programs.

## Conflict of interest

The authors declare no conflict of interest.
